# Differentials in the prevalence of anemia among non-pregnant, ever-married women in Bangladesh: multilevel logistic regression analysis of data from the 2011 Bangladesh Demographic and Health Survey

**DOI:** 10.1186/s12905-015-0211-4

**Published:** 2015-07-29

**Authors:** Md. Kamruzzaman, Md. Golam Rabbani, Aik Saw, Md. Abu Sayem, Md. Golam Hossain

**Affiliations:** Department of Statistics, University of Rajshahi, Rajshahi, 6205 Bangladesh; Department of Orthopaedic Surgery, National Orthopaedic Centre of Excellence for Research and Learning (NOCERAL), University of Malaya, Kuala Lumpur, Malaysia; Divisional TB Expert-Rangpur, National Tuberculosis Control Program, Directorate General of Health Services, Dhaka, Bangladesh

**Keywords:** Anemia, Bangladesh, Married women, Multilevel logistic regression

## Abstract

**Background:**

Anemia is one of the most common public health problems globally, and high prevalence has been reported among women of reproductive age, especially in developing countries. This study was conducted to evaluate differentials in the prevalence of anemia among non-pregnant, ever-married women of reproductive age in Bangladesh, and to examine associations with demographic, socioeconomic, and nutritional factors.

**Methods:**

Data for this cross-sectional study were taken from the 2011 Bangladesh Demographic and Health Survey (BDHS). In a sub-sample of one-third of the households, all ever-married women of reproductive age (15 to 49 years) were selected for the biomarker component of the survey, including anemia. The sample size for our study was 5,293. Data were analyzed using multilevel logistic regression analysis.

**Results:**

The prevalence of anemia among non-pregnant, ever-married women was 41.3 % (urban: 37.2 % and rural: 43.5 %). Among anemic women, 35.5 % had mild anemia, 5.6 % had moderate anemia, and 0.2 % had severe anemia. Women with no education were more likely to be anemic than those with secondary education (p < 0.01) or higher education (p < 0.01). Undernourished women (BMI < 18.5) were at greater risk of anemia (p < 0.01) compared with normal women, overweight women, and obese women. Anemia was less pronounced among non-pregnant women using contraception (p < 0.05), Muslim women (p < 0.01), and women living in rich households (p < 0.01).

**Conclusions:**

The prevalence of anemia among non-pregnant, ever-married women in Bangladesh is high. Illiteracy, poverty, and undernutrition are contributing factors.

## Background

Anemia is one of the major health problems globally despite advances in medical science [1]. Anemia in women of reproductive age (15–49) has been associated with numerous morbidities including miscarriage [2], preterm delivery [3], placental abruption [4], and low birth weight [5]. It is also related to a higher risk of prenatal and maternal mortality [6–8]. Persons with anemia are more prone to infectious diseases [9] and have a lower capacity to perform physical work [10]. In many developing countries, married women perform physical work (cultivation of fields) in addition to caring for children and managing the domestic responsibilities of a household (obtaining water and fuel, cooking, cleaning, etc.). Because the ability of married women in developing countries to carry out these important roles and responsibilities is directly related to their health status, this study of the prevalence of anemia among non-pregnant, ever-married women aged 15–49 in Bangladesh provides useful information about the impact of this debilitating disease on the health and wellbeing of a major subgroup of the population. Because this subgroup makes up approximately 94 % of the population of ever-married women of reproductive age in Bangladesh [11], by extension, these results can be applied to the larger population group, *all ever-married women* of reproductive age in Bangladesh, and to the even larger population group, *all women* of reproductive age (because almost all adult women in Bangladesh are “ever-married”). Ultimately, these prevalence figures have implications for Bangladeshi men as well, but that topic is not covered here.

The prevalence of anemia in Bangladesh has been reported since the early 1960s through national nutrition surveys of women and children, using small population samples [12]. More recently, Helen Keller International (HKI) collaborated with the Institute of Public Health Nutrition (IPHN) of Bangladesh to conduct anemia surveys in 1997–98 and again in 2001; however, these surveys were limited to documenting anemia prevalence in rural areas [13, 14]. Then, in 2011 the National Institute of Population Research and Training (NIPORT) in Bangladesh (with technical assistance provided by ICF International) collected a nationally representative sample of data on the prevalence of anemia among ever-married women of reproductive age. This data-collection activity was part of the 2011 Bangladesh Demographic and Health Survey (BDHS) [15]. Because three of the eight Millennium Development Goals (MDGs) set up by the Government of Bangladesh are related to improvements in health [15], the authors decided to investigate the differentials in anemia prevalence among ever-married women of reproductive age, using data from the 2011 BDHS. Because pregnancy increases the likelihood of anemia in women, the study focused on non-pregnant, ever-married women of reproductive age, examining selected socio-demographic factors associated with the prevalence of anemia in this large population group.

## Methods

Data for this cross-sectional study of anemia prevalence among ever-married women in Bangladesh come from the 2011 Bangladesh Demographic and Health Survey (BDHS), a nationally representative survey conducted from July 8, 2011 to December 27, 2011. The data set used in the analysis was obtained from NIPORT. The BDHS used a two-stage stratified cluster sampling method based on enumeration areas (EAs) and household samples. In the first stage, 600 EAs were selected with probability proportional to the size. In the second stage, a total of 18,000 residential households were selected, with an average of 30 households per EA. Additionally, in a sub-sample of one-third of the households, all ever-married women of reproductive age (15 to 49 years) were selected for the biomarker component of the survey, including anemia. Blood was taken from 5902 ever-married women of reproductive age for measurement of hemoglobin levels, as indicators of anemia status. Most of the measurements (95 %) were reported as complete and credible [15]. The sampling technique, survey design, survey instruments, measuring system, quality control, ethical approval and subject consent for the 2011 BDHS have been described elsewhere [15]. After excluding pregnant women from the sample, statistical techniques were used to check the data set for outliers [16]. It was determined that 5293 non-pregnant, ever-married women aged 15–49 were available for the study sample. The mean age of the women was 30.78 ± 9.27 years.

### Measurement and definition of outcome variable

Anemia status was the outcome variable of this study. The standard method for diagnosing anemia is to measure the level of hemoglobin (Hb) in a person’s blood; low Hb levels indicate anemia. The HemoCue rapid testing method was used in the 2011 BDHS to measure Hb levels. The HemoCue system consists of a battery-operated photometer and a disposable microcuvette (a small transparent vessel coated with a dried reagent that serves as the blood collection device). The microcuvette was used to transfer a drop of capillary blood from the woman’s fingertip to the photometer. The photometer analyzes the blood and reports the level of Hb (g/dl). The Hb level was adjusted for altitude and smoking status, according to the formulas recommended by the CDC. The definition of anemia and the process of blood testing in the BDHS are described in greater detail in the survey final report [15].

We first classified our sample into two groups: anemic women (Hb level < 12.0 g/dl) and non-anemic women (Hb level ≥12.0 g/dl). Anemic women were then subdivided into three subgroups – mild anemia (Hb level 10.0 to 11.9 g/dl), moderate anemia (Hb level 7.0 to 9.9 g/dl), and severe anemia (Hb level <7.0 g/dl) [11].

### Independent variables

The socioeconomic, demographic, and household information included in our study came from two of the five questionnaires used in the 2011 BDHS – the woman’s questionnaire and the household questionnaire. The questionnaires consisted of both structured (pre-coded) and non-structured (open-ended) questions. Body height and weight were measured and the body mass index (BMI) was calculated: ratio of weight in kilograms to height in meters squared (kg/m^2^).

The independent variables used in the study were: residence (urban/rural); woman’s education (none, primary, secondary, higher); husband’s education (none, primary, secondary, higher); breastfeeding (no/yes); currently amenorrheic (no/yes); currently using contraception (no/yes); currently working (no/yes); source of drinking water (non-improved/improved); toilet facility (unhygienic/hygienic); living with husband (no/yes); religion (non-Muslim/Muslim); wealth index (poor, middle, rich); mean BMI (thin [<18.5], normal [18.5–24.9], overweight [25.0–29.9], obese [≥30.0]); age group (15–29/30–49 years); age at first marriage (<18/18+ years); and children ever born (none, 1–2, 3–6, 7+). More detail on the definition of these variables is available in the 2011 BDHS survey report [15].

### Statistical analysis

Data used in this study were collected using multistage stratified clustered sampling; the dependence among observations comes from several levels of hierarchy. There is a cluster effect to the data set. A single-level statistical model would not be appropriate for analyzing this type of data set [17]. Instead, we used the intra-class correlation coefficient,

$$ \mathrm{I}\mathrm{C}\mathrm{C}=\frac{Variationof\  cons \tan t}{Variationof\  cons \tan t+\mathrm{v}\mathrm{a}\mathrm{r} iation\  of\  residual}. $$

The ICC was used initially to determine whether multilevel analysis was even necessary for the data. The value of ICC ranges from 0 to 1. If the ICC is 0, observations within clusters are not similar to observations from different clusters, and if the ICC is greater than 0, a multilevel regression model is appropriate for the analysis [18]. To remove the cluster effect, two levels of multiple logistic regression analysis were used for examining the association between anemia and socioeconomic, demographic and nutritional factors among ever-married women. The severity of anemia was the dependent variable. The multilevel logistic regression model is a powerful statistical tool for 1) removing the cluster effect, and 2) detecting an association between dependent (category) and independent variables at different levels of the data hierarchy. The two levels of multiple logistic regression models used in the study are:

**Level I:***η*_*ij*_ = *β*_0*j*_ + *β*_1*j*_*x*_*ij*_, ${P}_{ij}=\frac{ \exp \left({\eta}_{ij}\right)}{1+ \exp \left({\eta}_{ij}\right)}$ where *y*_*ij*_ = 1 with probability *P*_*ij*_, *y*_*ij*_ = 0, with probability 1-p_ij_,

$$ 1\mathrm{n}\left(\frac{P_{ij}}{1-{P}_{ij}}\right)={\beta}_{0j}+{\beta}_{1j}{x}_{ij} $$

**Level II:***β*_0*j*_ = *y*_00_ + *u*_0*j*_, *β*_1*j*_ = *y*_10_, *u*_0*j*_ ∼ *N*(0, *τ*_00_), $\pi =P\left(Y=1\left|{X}_1={x}_1,{X}_2={x}_2,\dots, {X}_p={x}_p\right.\right)=\frac{e^{g\left({x}_i\right)}}{1+{e}^{g\left({x}_i\right)}}$, where *g*(*x*_1_) = *β*_0_ + *β*_1_*x*_*i*1_ + *β*_2_*x*_*i*2_ + … + *β*_*k*_*x*_*ik*_; (*i* = 1, 2, …, *n*).

β_i_ = unknown logistic regression coefficients (i = 1, 2, …, n).

The parameter β_i_ refers to the effect of X_i_ on the log odds such that Y = 1, controlling the other X_i_. An important assumption in the multiple logistic regression model is that the explanatory variables are independent of each other (multicollinearity problem). In the present study, the magnitude of the standard error (SE) was used to detect the multicollinearity problem; if the magnitude of the SE lies between 0.001 and 0.5, there is no evidence of multicollinearity [19]. The Chi-square test was used for selecting independent factors for multilevel logistic regression models. Statistical significance was accepted at p < 0.05. Statistical analyses were carried out using STATA (version 11) and SPSS software (version IBM 19).

## Results

A total of 5293 non-pregnant, ever-married Bangladeshi women aged 15 to 49 were included in the study. The overall prevalence of anemia among these women was 41.3 % (urban: 37.2 % and rural: 43.5 %). Among anemic women, 35.5 % had mild anemia (urban: 31.9 % and rural: 37.5 %); 5.6 % had moderate anemia (urban: 5.2 % and rural: 5.9 %); and 0.2 % had severe anemia (urban: 0.1 % and rural: 0.2 %) (Table 1).

**Table 1:** Prevalence of anemia among non-pregnant, ever-married women aged 15–49, Bangladesh 2011

	Total % (N)	Urban % (N)	Rural % (N)
Anemia^a^			
Yes	41.3 (2186)	37.2 (690)	43.5 (1496)
No	58.7 (3107)	62.8 (1166)	56.5 (1941)
Type of anemia^b^			
Mild	35.5 (1880)	31.9 (592)	37.5 (1288)
Moderate	5.6 (298)	5.2 (96)	5.9 (202)
Severe	0.2 (8)	0.1 (2)	0.2 (6)

^a^Anemia (Hb level < 12.0 g/dl); no anemia (Hb level ≥12.0 g/dl)

^b^Mild anemia (Hb level 10.0 to 11.9 g/dl); moderate anemia (Hb level 7.0 to 9.9 g/dl); severe anemia (Hb level <7.0 g/dl) [11]

The Chi-square (*χ*^2^) test was used to investigate the association between anemia and selected factors (Table 2). Comparing anemia prevalence by residence, we found that anemia was much higher among women living in rural areas (43.5 %) than those living in urban areas (37.2 %), and the association was statistically significant (p < 0.001). By level of education, anemia prevalence was 45.9 % for women with no education, 43.0 % for those with primary education, 38.1 % of those with secondary education, and 33.2 % for those with higher education. The association between level of education and anemia was statistically significant (p < 0.001) for both women and their husbands.

**Table 2:** Association between anemia and demographic and socioeconomic variables, non-pregnant, ever-married women aged 15–49, Bangladesh 2011

Variable	Percentage with anemia^a^	*χ* ^2^-value	*p*-value**	No. of women
Residence		20.043	*p* < 0.001	
Urban	37.2			1856
Rural	43.5			3437
Education				
No education	45.9	32.940	*p* < 0.001	1402
Primary	43.0			1620
Secondary	38.1			1873
Higher	33.2			398
Husband’s education				
No education	44.7	20.965	*p* < 0.001	1546
Primary	43.0			1498
Secondary	38.5			1492
Higher	36.6			757
Breastfeeding				
No	39.7	16.293	*p* < 0.001	3935
Yes	45.9			1358
Currently amenorrheic				
No	40.4	28.123	*p* < 0.001	5003
Yes	56.2			290
Currently using contraceptive method				
No	44.1	10.598	*p* < 0.001	2025
Yes	39.6			3268
Currently working				
No	40.9	1.898	0.1680	4550
Yes	43.6			743
Drinking water source				
Non-improved	41.0	0.013	0.9090	458
Improved	41.3			4835
Toilet facility				
Unhygienic	43.3	7.871	*p* < 0.01	2467
Hygienic	39.5			2826
Living with husband				
No	43.4	0.701	0.4030	350
Yes	41.1			4943
Religion				
Muslim	40.2	19.370	*p* < 0.001	4668
Non-Muslim	49.4			625
Wealth Index				
Poor	47.5	57.348	*p* < 0.001	1891
Middle	42.1			1016
Rich	36.0			2386
Body Mass Index				
Thin	50.5	94.302	*p* < 0.001	1302
Normal	40.8			3035
Overweight	31.0			788
Obese	27.4			168
Age group				
15–29 years	38.6	13.579	*p* < 0.001	2411
30–49 years	43.6			2882
Age at first marriage				
Less than 18 years	41.9	2.505	0.1130	4137
18 years and older	39.3			1156
Number of children ever born				
None	37.7	27.545	*p* < 0.001	422
1–2	38.4			2411
3–6	44.1			2219
7 or more	51.0			241

Note: ^a^Anemia (Hb level < 12.0 g/dl), **p-value (*p* < 0.001, 0.1 % level of significance)

The prevalence of anemia was higher among women who were currently breastfeeding (45.9 %) than among non-breastfeeding women (39.7 %), and the associations were statistically significant (p < 0.001). The percentages of women with anemia were higher among those who were currently amenorrheic and those who were not using contraception; the association of these two factors and anemia was significant (p < 0.001).

Women living in households with unhygienic toilets were more likely to be anemic compared with women living in households with hygienic toilets, and the association was statistically significant (p < 0.01). Anemia was more common among non-Muslim women (49.2 %) than among Muslim women (40.2 %), and women living in poor households (47.5 %) and middle-income households (42.1 %) were more likely to be anemic compared with those living in rich households (36.0 %). All these associations were statistically significant (p < 0.001). Low BMI (<18.5) in women was closely associated with anemia. More than 50 % of the non-pregnant, ever-married women in this subgroup were anemic, compared with 40.8 % in the normal group, 31.0 % in the overweight group, and 27.4 % in the obese group. As expected, the association between BMI and anemia prevalence was statistically significant (p < 0.001).

Looking at differentials by age group, we noted that older women (30–49 years) were more likely to be anemic than younger women (15–29 years), 43.6 % and 38.6 %, respectively; the association was statistically significant (p < 0.001). Women with seven or more children had a higher prevalence of anemia (51.0 %) than women with three to six children (44.1 %), women with one or two children (38.4 %), and women with no children (37.7 %). The association between parity and anemia was statistically significant (p < 0.001). Variables for which no significant association with anemia was found included: currently working, source of drinking water, living with husband, and age at first marriage (p > 0.05).

All the associated factors were regarded as independent variables for anemia in the two-level logistic regression model. Before using this model, we calculated the value of the intra-class correlation coefficient (ICC) within the clusters. The value of the ICC was found to be 0.063; therefore, the multilevel model was considered appropriate for the study. The model demonstrated that women living in rural areas were more likely to have anemia than those living in urban areas (OR = 0.854, 95 % CI: 1.004–0.726; p = 0.057). Women with no education were more likely to be anemic than those with secondary education (OR = 0.769, 95 % CI: 0.919–0.644; p < 0.01) or higher education (OR = 0.638, 95 % CI: 0.870–0.468; p < 0.01). Thin women (BMI < 18.5) had a greater chance of being anemic than normal women (OR = 0.717, 95 % CI: 0.827–0.622; p < 0.01), overweight women (OR = 0.491, 95 % CI: 0.605–0.368; p < 0.01), or obese women (OR = 0.396, 95 % CI: 0.584–0.268; p < 0.01).

Looking at the rest of the variables included in the analysis, women were more likely to become anemic than their counterparts if they were: non-Muslim (OR = 1.506, 95 % CI: 1.238–1.833; p < 0.01), not currently using contraception (OR = 0.875, 95 % CI: 0.999–0.766; p < 0.05), currently breastfeeding (OR = 1.349, 95 % CI: 1.593–1.143; p < 0.01), currently amenorrheic (OR = 1.635, 95 % CI: 2.197–1.217; p < 0.01), or in the age group 30–49 years (OR = 1.469, 95 % CI: 1.723–1.253; p < 0.01). Women who lived in poor households were more likely to be anemic than women who lived in rich households (OR = 0.781, 95 % CI: 0.918–0.664; p < 0.01) (Table 3).

**Table 3:** Effects of demographic and socioeconomic factors on anemia using multilevel logistic regression, non-pregnant, ever-married women aged 15–49, Bangladesh 2011

Variable	Coefficients	SE	*p*-value*	Odds Ratio (OR)	95 % CI of OR	
					Upper	Lower
Residence	−0.0349	0.0184	0.057	0.854	1.004	0.726
Urban (Ref. Rural)						
Education Level						
Primary (Ref. No)	−0.0248	0.0184	0.179	0.897	1.053	0.765
Secondary (Ref. No)	−0.0591	0.0204	*p* < 0.01	0.769	0.919	0.644
Higher (Ref. No)	−0.0988	0.0347	*p* < 0.01	0.638	0.870	0.468
Husband’s Education						
Primary (Ref. No)	0.0108	0.0185	0.559	1.049	1.233	0.894
Secondary (Ref. No)	−0.0112	0.0206	0.585	0.950	1.137	0.795
Higher (Ref. No)	0.0120	0.0283	0.671	1.055	1.353	0.823
Type of toilet at home,	−0.0229	0.0138	0.099	0.903	1.019	0.799
Unhygienic (Ref. Hygienic)						
TV watching	−0.0003	0.0164	0.987	0.999	1.156	0.865
Yes (Ref. No)						
Religion	0.9338	0.0227	*p* < 0.01	1.506	1.833	1.238
Non-Muslim (Ref. Muslim)						
Currently contraceptive use	−0.2925	0.0148	*p* < 0.05	0.875	0.999	0.766
Yes (Ref. No)						
Currently breastfeeding	0.0649	0.0185	*p* < 0.01	1.349	1.593	1.143
Yes (Ref. No)						
Currently amenorrheic	0.1118	0.0334	*p* < 0.01	1.635	2.197	1.217
Yes (Ref. No)						
Wealth Index						
Middle (Ref. Poor)	−0.03334	0.0194	0.086	0.865	1.027	0.729
Rich (Ref. Poor)	−0.0551	0.0183	*p* < 0.01	0.781	0.918	0.664
Age Group	0.0832	0.0176	*p* < 0.01	1.469	1.723	1.253
(30–49) years (Ref. 15–29 years)						
BMI category						
Normal (Ref. Thin)	−0.0759	0.0162	*p* < 0.01	0.717	0.827	0.622
Overweight (Ref. Thin)	−0.1559	0.0232	*p* < 0.01	0.491	0.605	0.368
Obese (Ref. Thin)	−0.1991	0.0407	*p* < 0.01	0.396	0.584	0.268
Number of children ever born						
1–2 (Ref. No)	−0.0215	0.0271	0.426	0.901	1.149	0.706
3–6 (Ref. No)	0.0007	0.0293	0.980	0.995	1.294	0.764
7+ (Ref. No)	0.0282	0.0426	0.508	1.117	1.629	0.766
Constant	0.4849	0.0198	*p* < 0.01			

Note: *p-value (*p* < 0.01, 1 % and *p* < 0.05, 5 % level of significance)

## Discussion

In this study we examined differentials in the prevalence of anemia among non-pregnant, ever-married women in Bangladesh in relation to selected socioeconomic and demographic factors. Previous studies in Bangladesh have reported on anemia among women living in rural areas [20], university students in a specific region [21], infants [22], and students attending a medical college [23]. Using multilevel regression analysis, we were able to minimize the cluster effect of the data set from the 2011 BDHS [15]. Therefore, our results provide a broader, more representative picture of this important population group.

The prevalence of anemia in our study population of non-pregnant, ever-married women in Bangladesh (41.3 %) is higher than levels reported in many other countries around the world (developed and developing) including: Belgium, 7.7 % [24], Brazil, 32.7 % [25], China, 15.1 % [26], Iran, 14.5 % [27], Japan, 15.7 % [28], Kazakhstan, 39 % [29], Mexico, 15.5 % [30], Serbia, 27.7 % [31], and Turkey, 32.8 % [32]. Globally, the prevalence of anemia is 29 % [33]. The level of anemia among Bangladeshi women is similar to that found in some countries in west and central Africa, 40 % [34]. In only two countries – India, 56 % [35] and Tanzania, 49 % [36] – was the prevalence of anemia higher than in Bangladesh.

It is clear that the general health status of ever-married women in Bangladesh requires substantial improvement. It is suggested that the factors associated with higher levels of anemia prevalence among non-pregnant, ever-married women in Bangladesh could function as a proxy index for general population health. Such an index might be useful for more effective targeting of vulnerable groups.

As noted earlier, the prevalence of anemia is higher among non-pregnant, ever-married women in rural areas (43.5 %) compared with those in urban areas; however, this level is considerably lower than that reported by the Bangladesh Bureau of Statistics (BBS) [37] for the 2003 census. In that year, 73 % of non-pregnant Bangladeshi women living in rural areas were reported to have anemia. A decline in the prevalence of anemia in rural areas between 2003 and 2011 may reflect an improvement in the general health status of women in rural areas, but the size of the decline (30 percentage points) seems overly large. The census also reported anemia prevalence of 34 % for women living in urban areas [37], a figure that is much closer to the prevalence found in our study for women living in urban areas (37.2 %).

Poverty and lack of education were two important factors related to the prevalence of anemia among Bangladeshi women. Household economic status (wealth index) is an important predictor of anemia, with women living in poor households more likely to be anemic compared with those living in middle or rich households. In developing countries where a skilled and trained work force is lacking, we would expect a higher level of education to be associated with socioeconomic benefits such as better nutrition and better health care. However, we found that even among the most educated women in the study, those with higher education, one-third (33.2 %) had anemia; this compares with almost half (46 %) of women with no education. The Bangladesh Government has taken steps to increase the literacy rate by adopting a national education policy [15] that provides free education through the secondary level. The government also provides subsidies for girls from poor families to attend school. Increasing the level of education will likely bring about a gradual decline in the prevalence of anemia.

In Bangladesh it is customary for a woman to marry a man of the same socioeconomic status. Thus, it is not surprising that the study showed that the husband’s level of education reflects that of the woman, as does the associated risk of anemia. The use of unhygienic toilets is an important factor in Bangladesh because of the increased risk of parasitic infestation in the tropical climate. The association of unhygienic toilets with anemia is explained by poor general health and/or chronic blood loss through gastrointestinal parasite infestation. The majority of the population of Bangladesh is Muslim. Non-Muslims, mostly Hindus and Buddhists, have a slightly higher prevalence of anemia than Muslims. It has been suggested that because many Hindus and Buddhists are vegetarians the higher levels of anemia in these groups may be associated with dietary preferences. Research is needed to clarify this issue.

Our study showed that undernourished women (BMI < 18.5) are more likely to have anemia than women whose BMI is normal, or women who are overweight or obese. A positive association between the level of Hb and body mass index (BMI) has been reported [38]. Women currently using a contraceptive method had a lower risk of anemia than those who were not using contraception; this finding has been reported in other studies [36, 39]. Lactating women were more likely to be anemic than non-lactating women, which is thought to be primarily due to increased nutritional demand during lactation [40]. This finding may also partially explain why women with higher parity were more likely to have anemia. Multiple periods of breastfeeding (from successive children) would tend to diminish the health status of higher-parity mothers, although there may be other contributing factors. For women with low iron or nutritional reserves, loss of blood through menses can contribute to anemia. In our study, however, amenorrheic women were more likely to have anemia than their counterparts. While we do not have adequate information to comment on this counterintuitive finding, amenorrhea is a multi-factorial condition that may include undiagnosed pregnancy, prolonged lactation and poor general health. In Bangladesh, women aged 30 to 49 generally have young children who are dependent on them for basic needs such as food, shelter, and care. Married women, who are typically both mothers and wives, often put the needs of the household/ family/husband above themselves, which may result in poor nutritional status and anemia.

## Conclusion

The prevalence of anemia among non-pregnant, ever-married women of reproductive age in Bangladesh is higher than that of their counterparts in most developing countries. Women living in rural areas, those with no education, and women in poor households have significantly higher rates of anemia. Other variables associated with anemia are: being non-Muslim, being undernourished (BMI < 18.5), not currently using a contraceptive method, currently breastfeeding, amenorrheic, and being aged 30 to 49. Improving the level of education and economic status of women, particularly in rural areas, would contribute to reducing the prevalence of anemia in this population.

## Abbreviations

BBS: Bangladesh Bureau of Statistics
BDHS: Bangladesh Demographic and Health Survey
BMI: Body mass index
CDC: Centers for Disease Control and Prevention
EA: Enumeration area
HKI: Helen Keller International
ICC: Intra-class correlation coefficient
IPHN: Institute of Public Health Nutrition
MDGs: Millennium Development Goals
